# New Lunacy Legislation

**Published:** 1890-05

**Authors:** 


### NEW LUNACY LEGISLATION.

CHAPTER 283, of the Laws of 1889, contains a section of interest to the general practitioner, to which we desire to call attention. It is the Act establishing a Commission in Lunacy for this State, and Section 7 reads as follows:

*Section 7.* "The said Commission shall keep in its office records, showing the names and residences of all Judges in this State, who are empowered by law to approve medical certificates of insanity, or to make an order of commitment of an insane person to custody; and also a record showing the name, residence and certificate of each medical examiner in lunacy qualified in accordance with the laws of this State; and it is hereby made the duty of each medical examiner in lunacy, at the time of the passage of this Act, to forward to the State Commission in Lunacy, a certified copy of his certificate of qualification."

"Hereafter, it shall be the duty of every physician who receives a certificate as a medical examiner in lunacy in this State, to forward a certified copy thereof to the office of the commission within three days after such certificate is granted, and said commission shall cause the same certificate to be recorded as soon as received, and shall promptly advise said physician of the recording thereof in writing. *One year after the date of the passage of this Act, it shall not be lawful for any medical examiner in lunacy to make a certificate of insanity for the purpose of committing any person to custody, unless his certificate has been so forwarded, and its record in the office of the commission, as above provided, has been acknowledged.*"

The latter clause of this section (italicized) is the one to which we particularly refer. The year expires on the 14th day of May, 1890, and after this date certificates of lunacy cannot legally be made except by those who have met the requirements of the law, by having sent a copy of their certificates of qualification to the Commission in Lunacy and having received from them an acknowledgment that their names have been placed on file in that office as qualified examiners.

This is a very simple act, but the law makes it an important one, as upon its performance depends the legality of the commitment of a patient to an asylum.

Soon after the passage of the law, the Commission in Lunacy sent to the practitioners of the State copies of the act, that they might see what was demanded of them. Some physicians have already taken the necessary steps to make themselves legally qualified examiners, and we would urge upon all the necessity of this action.

Dr. E. H. Howard, Superintendent of the Monroe County Asylum, called attention to the subject in a paper read before the State Medical Society in February last, in which he pointed out the difficulties that would arise from neglect to comply with this provision of the law.

It is made the duty of Superintendents of Asylums to forward promptly to the Commission, copies of the medical certificates in every case committed to the institution under their charge. If the medical examiners neglect their duty of being registered at Albany, the patient cannot be legally committed nor detained.

This neglect will cause infinite trouble and give rise to many legal complications to the great annoyance of the physicians who act as examiners, as well as to the officers of the asylums and the friends of patients.

We bring this to the notice of members of the profession, as there may be some who are ignorant of the law, and others who have neglected to meet its requirements. Let every qualified physician at once send to the Commission in Lunacy, whose office is in the State Capitol, at Albany, N. Y., copies of their certificates of qualification. They will then be prepared at any time to make certificates for the commitment of their patients to custody.

It is the duty of the Judge approving the certificates to assure himself in each case that the medical man making the certificate is legally qualified and has met this new requirement of the law.

We would recommend that each physician file in the County Clerk's office, his certificate of qualification and obtain a statement to that effect, which can then be forwarded to Albany. The Clerk of Erie County has prepared the proper form for filing in his office and for transmission to the Commission in Lunacy.

It is gratifying to note that it is announced that Governor Hill has signed the bill lately passed, rechristening the insane asylums throughout the State. They are insane asylums no longer, but State Hospitals. The Buffalo State Hospital will be hereafter the official designation of the institution in this city presided over by Dr. J. B. Andrews.

